# Multiscale Characteristics and Drivers of the Bundles of Ecosystem Service Budgets in the Su-Xi-Chang Region, China

**DOI:** 10.3390/ijerph191912910

**Published:** 2022-10-09

**Authors:** Yue Wang, Qi Fu, Tinghui Wang, Mengfan Gao, Jinhua Chen

**Affiliations:** 1 School of Politics and Public Administration, Soochow University, Suzhou 215123, China; 2 The Institute of Regional Governance, Soochow University, Suzhou 215123, China; 3 Research Institute of Metropolitan Development of China, Soochow University, Suzhou 215123, China

**Keywords:** ecosystem services, budget, multiscale, drivers, rapid urbanization

## Abstract

Managing ecosystem services (ESs) to meet human needs is critical to achieving sustainable development in rapidly urbanizing regions. Identifying ES budget bundles and analyzing their drivers at a multiscale level can facilitate management decision-making; however, further research is required in areas undergoing rapid urbanization. This study quantified the supply, demand, and budgets of six typical ESs at the county, township, and village scales in the Su-Xi-Chang region in 2020. Additionally, the influence of natural environmental and socioeconomic factors on ES budget bundles was investigated based on K-means cluster analysis and the Geodetector model. The results showed that ESs on all three scales showed a mismatch between supply and demand. The similarity in the spatial pattern of supply, demand, and budgets of ESs at the township and village scales was higher than that at the township and county scales. The location and area of surplus, balance, and deficit varied with scale. We found that population density and the proportion of impervious surfaces are the main factors influencing the formation of the ES budget bundles at different scales. In addition, the diversity and degree of interpretation of drivers varied with scale. We believe that focusing on the overall situation on a large scale and implementing precise management on a small scale can make management decisions more effective. This study can provide a scientific basis for the sustainable utilization of ESs in the Su-Xi-Chang region, and the research results and methods can provide a reference for similar studies in other rapidly urbanizing areas in the world.

## 1. Introduction

Ecosystem services (ESs) refer to the various benefits humans directly or indirectly derive from ecosystems [1]. Trees, grasslands, and water systems in cities provide residents with multiple important ESs. By 2050, 68% of the world’s population is expected to live in cities [2]. Urban ecosystem services will be more closely linked to human well-being [3]. However, with the acceleration of urbanization, 63% of global ESs have degraded sharply [4]. The reduction in ESs will constrain economic development and affect human well-being.

The supply and demand of ES linking natural ecosystems with socioeconomic systems have been a research hotspot in the past decade [5,6,7]. The supply of ESs is the products and services that the ecosystem provides to humans [8]. The demand for ESs is the preferential consumption and use of products and services provided by ecosystems by humans [9]. The evaluation methods of supply are mainly the model simulation method [10] and the value evaluation method [1,11]. Due to easy access to input parameters and accurate evaluation results, the Integrated Valuation of Ecosystem Services and Tradeoffs (InVEST) developed by the Natural Capital Project has become one of the most commonly used models for evaluating ES supply. It can be used to quantitatively assess various ESs, such as flood mitigation, urban cooling, water yield, and carbon sequestration. The assessment methods of demand are mainly based on the LULC matrix method [12], questionnaire survey method [13], and ecological footprint method [14]. The supply of ES is determined by ecosystem components, structures, and processes [15]. For example, forest ecosystems can provide considerable regulation services and cultural services, while farmland ecosystems are more capable of providing provisioning services [16]. The demand for ESs is related to the preferences of different stakeholders [9]. For example, residents are more concerned about quick-profit entertainment services [13,17] and provisioning services, such as water [18] and food [19]. Policy-makers favor regulation services with long-term benefits, such as flood regulation [20], water purification [21], and carbon sequestration [18]. This leads to an imbalance in the distribution of ES supply and demand with strong spatial heterogeneity [22]. In highly urbanized areas, the spatial heterogeneity of ES supply and demand often manifests as an imbalance or mismatch [3]. The reduction in ES supply further exacerbates the supply–demand imbalance [23]. This imbalance will become more severe as urbanization accelerates [18]. Coupled with ES supply and demand research, it is possible to understand the gap between supply and demand and determine the position of deficit (supply is less than demand) to achieve precise management [24].

The formation, consumption, and management of ESs are scale-dependent [25,26]. Findings on a single scale often cannot be directly applied to other scales; usually, 2-4 scales will give better results [27]. However, most studies only observe ES supply and demand at a single scale, such as a certain administrative region scale [13] or watershed scale [28], which may not fully grasp the connection between ES supply and demand [24]. Multiscale research has shifted from focusing on trade-offs and synergies between services [29,30] to gradually focusing on supply–demand correlation research and management decision-making [3,22,31]. A large number of studies have shown that applying the multiscale analysis perspective of ES to the decision-making process can avoid problems such as a lack of management measures and inefficiency caused by scale mismatch [31,32,33,34].

Multiscale analysis combines the advantages of large and small scales. The large scale can understand the overall situation of ES supply and demand and determine the main position of supply–demand imbalance, and the small scale can understand the local situation and implement accurate management [35]. With the development of 3S technology, ES spatial mapping provides clearer and more accurate information. However, multiscale studies mostly focus on large scales, such as urban agglomerations [36] and provinces [22]. Focusing on small scales, such as townships and villages, with the support of more detailed data is needed to further strengthen multiscale research [31].

Understanding the interactions between ESs can help with scientific management decisions [37]. Ecosystem service bundles (ESBs) are a combination of ESs that recurs in a certain space or time [38]. A set of ESBs reflects similar social–ecological characteristics in different landscape regions. The current research type is dominated by ES supply bundles [39,40]. The identification methods are mainly K-means and hierarchical clustering analysis [41]. ESBs are formed in global [42], European [39], national [43], watershed [40], and local regions [44]. This suggests that ESBs do not appear randomly but are influenced by socioeconomic [42,43] and natural environment factors [4,44,45]. Methods to identify drivers of ESB include principal component analysis [46], the Geodetector model [4], and random forests [47]. In addition, ESB manifests differently at different scales, and researching only one scale will omit or distort the relationship between ESs at other scales [25]. It is important to explore ES budget bundles and their driving factors from a multiscale level, but further research is required in regions undergoing rapid urbanization.

The Su-Xi-Chang region is typically representative of an area undergoing rapid urbanization in China. Its urbanization driving force comes from the development of rural industrialization, and it is a representative area of bottom-up urbanization in China. The three cities in the region—Suzhou, Wuxi and Changzhou—have similar characteristics in terms of their social culture, industrial layout and ecological structure, which are also typical among many metropolitan areas in China. However, due to the rapid economic development in recent years, areas under construction have occupied a large amount of cultivated land and water, resulting in a loss of ESs such as food production, water yield, landscape recreation, and climate regulation [48,49]. However, people’s demand for natural resources and a better living environment is increasing, which makes the contradiction between people and land in the Su-Xi-Chang region more prominent. In addition, in the “Yangtze River Delta Urban Agglomeration Development Plan (2015–2030)”, it is also proposed that the Su-Xi-Chang region should speed up the restoration of ecological space. Therefore, it is urgent to understand the location and drivers of ES supply and demand imbalance to make management decisions.

To narrow the research gaps, we researched the supply and demand of ES in the Su-Xi-Chang region from a multiscale level. First, we quantitatively evaluated the supply, demand, and budgets of six typical ESs (crop production (CP), water retention (WR), PM_2.5_ reduction (PR), heat mitigation (HM), flood mitigation (FM), and landscape recreation (LR)) at the county, township, and village scales in 2020. Second, we identified the spatial pattern of ES budget bundles at three scales using k-means clustering analysis. Third, we used the Geodetector model to identify natural environmental and socioeconomic factors that influence the formation and spatial distribution of ES budget bundles at different scales. This paper aims (1) to reveal the multiscale pattern characteristics of supply, demand, and budgets in typical urban ESs in the Su-Xi-Chang region and (2) to identify spatial heterogeneity and scale dependence of ES budget bundles and their drivers. This study can provide a scientific basis for the sustainable utilization of ecosystem services in the Su-Xi-Chang region. The research results and methods can provide a reference for similar studies in other rapidly urbanizing areas in the world.

## 2. Materials and Methods

### 2.1. Study Area

**Figure 1:**
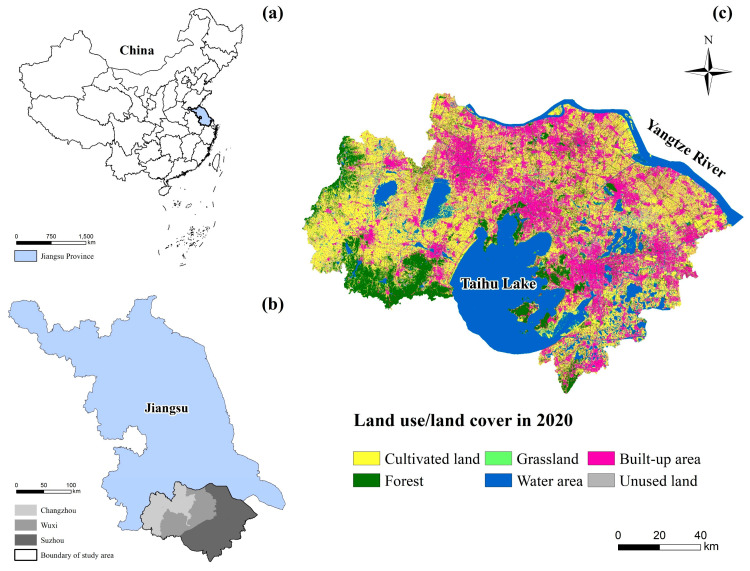
(**a**) The location of Jiangsu province in China; (**b**) the location of the Su-Xi-Chang region in Jiangsu province; (**c**) the land use/land cover (LULC) pattern of the Su-Xi-Chang region in 2020.

The Su-Xi-Chang region (119°08′–121°15′ E and 30°46′–32°04′ N) is located in the southern part of Jiangsu Province, China, including three cities of Suzhou, Wuxi, and Changzhou (Figure 1a,b). The total area is about 17,700 square kilometers. The Su-Xi-Chang region borders Shanghai to the east, Zhejiang to the south, and the Yangtze River to the north. The terrain of the study area is flat, and the average elevation is below 50 m. The area is in a subtropical monsoon climate zone, with an average annual temperature of 15.1–17.3 °C and average annual precipitation of 1093 mm. The vegetation in the area is mainly subtropical evergreen, broad-leaved forest. The land use types are mainly cultivated land (31.32%), built-up area (23.1%), and water (22.56%) (Figure 1c). The lakes include Taihu Lake, Yangcheng Lake, and Gehu Lake.

Since the early 1980s, Shanghai, the most important financial center in China, has brought the Su-Xi-Chang region into an industrial age. In the mid-1980s, a development model for realizing rural in situ urbanization through the development of township enterprises appeared in the Su-Xi-Chang region, called the “South Jiangsu Model” [50]. After 2000, the Su-Xi-Chang region entered a stage of rapid urbanization [49]. By 2020, the total population of the Su-Xi-Chang region reached 25.49 million. The average population density (1439 persons/km^2^) was 9.73 times the national population density (148 persons/km^2^). The per capita GDP (158,263 CNY) was 2.20 times the national average (72,000 CNY). The urbanization rate (81.07%) is 1.29 times the national urbanization rate (63.89%). Population growth, economic development, and rapid industrialization have changed the local land use structure [51]. This is mainly manifested in the increased demand for construction land; the occupation of farmland, water bodies, and wetlands along with landscape fragmentation, ES loss, and other problems, which seriously affect the sustainable development of the ecosystem in the Su-Xi-Chang region [49,52,53].

### 2.2. Quantification of Supply and Demand for ESs

Combining the importance to the lives of local residents, the feasibility of assessment methods and data, and referring to the study of [31,54,55,56,57,58], we selected six typical ESs (crop production (CP), water retention (WR), PM2.5 reduction (PR), heat mitigation (HM), flood mitigation (FM), and landscape recreation (LR)) as research objects and simulated their supply and demand. The required data are shown in Table 1. The resolution of the raster data in this study is uniformly set to 30 m × 30 m, and the projected coordinate system is WGS_1984_UTM_Zone_51N. The LULC type is divided into six categories: cultivated land, forest, grassland, water area, built-up area, and unused land.

#### 2.2.1. Crop Production

(1) Supply

Referring to the method of [60], the crop production was distributed to each pixel according to the ratio of the Vegetation Condition Index (VCI) of each cultivated land pixel. The calculation is as follows:

(1) $${\mathrm{CP}}_{i}={\mathrm{CP}}_{s}\times \frac{{\mathrm{VCI}}_{i}}{\sum_{i=1}^{N}{\mathrm{VCI}}_{i}}$$

(2) $${\mathrm{VCI}}_{i}=\frac{\left({\mathrm{NDVI}}_{i}-{\mathrm{NDVI}}_{\min}\right)}{{\mathrm{NDVI}}_{\max}-{\mathrm{NDVI}}_{\min}}\times 100\%$$

${\mathrm{CP}}_{i}$ is the crop production of the ith cultivated land pixel, t; ${\mathrm{GP}}_{s}$ is the total crop production in the Su-Xi-Chang region in 2020, ${\mathrm{GP}}_{s}\;$= 2.09 × 106 t; N is the total number of pixels of cultivated land in the study area; ${\mathrm{NDVI}}_{i}$ is the annual NDVI value of the ith cultivated pixel; and ${\mathrm{NDVI}}_{\max}$ and ${\mathrm{NDVI}}_{\min}$ represent the maximum and minimum annual NDVI values of the cultivated land in the Su-Xi-Chang region.

(2) Demand

We defined the demand for crop production (CP) by crop consumption, mainly including wheat and grains. The per capita annual crop consumption comes from the 2020 Jiangsu Statistical Yearbook. The calculation formula is:

(3) $$D_{\mathrm{ci}}=P_{\mathrm{ci}}\times C_{a}$$

$D_{\mathrm{ci}\;}$ is the crop demand on pixel i, t; $P_{\mathrm{ci}}$ is the population on pixel i, person; and $C_{a}$ is the per capita annual crop consumption in the Su-Xi-Chang region, $C_{a}$ = 0.1221 t/person.

#### 2.2.2. Water Retention

(1) Supply

The supply of water retention (WR) is the difference between water yield and surface runoff [61]. Water yield was calculated using the InVEST Water Yield model. The model defines the water yield as the difference between precipitation and actual evapotranspiration. The plant’s available water content was calculated using the formula from [62]. The Biophysical Table refers to the study of [63]. The surface runoff coefficient refers to Guidelines for Delineation of Ecological Protection Red Lines. The calculation is as follows:

(4) $$Y_{\mathrm{ij}}=\left(1-\frac{{\mathrm{AET}}_{\mathrm{ij}}}{P_{i}}\right)\times P_{i}$$

(5) $$\frac{{\mathrm{AET}}_{\mathrm{ij}}}{P_{i}}=\frac{1+w_{i}R_{\mathrm{ij}}}{1+w_{i}R_{\mathrm{ij}}+\frac{1}{R_{\mathrm{ij}}}}$$

(6) $${\mathrm{WR}}_{\mathrm{ij}}=Y_{\mathrm{ij}}-{\mathrm{Runoff}}_{\mathrm{ij}}$$

(7) $${\mathrm{Runoff}}_{\mathrm{ij}}=P_{i}\times C_{j}$$

where $Y_{\mathrm{ij}}$ is the annual water yield of LULC type j on pixel i, mm; ${\mathrm{AET}}_{\mathrm{ij}}$ is the average annual actual evapotranspiration on pixel i, mm; $P_{i}$ is the average annual precipitation on pixel i, mm; $R_{\mathrm{ij}}$ is the Budyko Dryness Index on pixel i, dimensionless; $w_{i}$ is a non-physical parameter characterizing natural climate and soil properties, dimensionless; ${\mathrm{WR}}_{\mathrm{ij}}$ is the annual water retention of LULC type j on pixel i, mm; ${\mathrm{Runoff}}_{\mathrm{ij}}$ is the annual surface runoff on pixel i, mm; and $C_{j}$ is the average surface runoff coefficient of LULC type j. The specific coefficient values are shown in Table 2.

(2) Demand

This study expressed the demand for WR as the sum of agricultural, domestic, industrial, and ecological water consumption [24]. We assigned the average water consumption per unit area of cultivated land, industrial land, forest and grassland in each subdistrict to the cultivated land, industrial land, forest and grassland in each subdistrict to obtain the agricultural water consumption ($D_{\mathrm{ai}}$), industrial water consumption($D_{\mathrm{ii}}$), and ecological water consumption($D_{\mathrm{ei}}$) on pixel i. The assignment procedure was conducted using the analysis tool in ArcGIS 10.8 software. The calculation is as follows:

(8) $$D_{\mathrm{wi}}=D_{\mathrm{ai}}+D_{\mathrm{di}}+D_{\mathrm{ii}}+D_{\mathrm{ei}}$$

(9) $$D_{\mathrm{di}}=W_{c}\times P_{\mathrm{wi}}$$

(10) $$A_{a}=\frac{{\mathrm{CA}}_{\mathrm{subdistrict}}}{{\mathrm{SA}}_{\mathrm{subdistrict}}}$$

where $D_{\mathrm{wi}}$ is the annual water consumption on pixel i, m^3^; $D_{\mathrm{ai}}$, $D_{\mathrm{di}}$,${\;D}_{\mathrm{ii}}$, and $D_{\mathrm{ei}}$ are the agricultural water consumption, domestic water consumption, industrial water consumption, and ecological water consumption on pixel i, respectively, m^3^; $W_{c}$ is the per capita annual domestic water consumption in the Su-Xi-Chang region in 2020, m^3^/person; $P_{\mathrm{wi}}$ is the population on pixel i, persons; $A_{a}$ represents the average water consumption per unit of cultivated land; ${\mathrm{CA}}_{\mathrm{subdistrict}}$ represents the total water consumption of each subdistrict; ${\mathrm{SA}}_{\mathrm{subdistrict}}$ represents the cultivated land area of each subdistrict; and $D_{\mathrm{ii}}$ and $D_{\mathrm{ei}}$ are calculated in the same way as $D_{\mathrm{ai}}$.

#### 2.2.3. PM_2.5_ Reduction

(1) Supply

In this study, the supply of PM_2.5_ reduction (PR) was characterized by the amount of PM_2.5_ particles adsorbed by the forest, which was calculated using the dry deposition model [64]. The calculation is as follows:

(11) $$Q_{y}=D\times Q_{d}$$

(12) $$Q_{d}=F\times \mathrm{TCLA}\times T\times \left(1-R\right)$$

(13) $$F=V_{d}\times C_{h}\times 3600÷1000000$$

(14) $$\mathrm{TCLA}=T_{c}\times \mathrm{LAI}$$

where $Q_{y}$ is the total reduction in PM_2.5_ particles by a forest in one year, g; D is the number of non-rainy days in a year; $Q_{d}$ is the total reduction in PM_2.5_ particles by a forest in one day, g; F is the PM_2.5_ dry deposition flux, g/(m^2^·h); TCLA is the leaf surface area of different types of forest, m^2^; T is the evaluation time, the number of hours per day, T = 24; R is the resuspension rate, R = 0.03; $V_{d}$ is the deposition velocity, which is related to the wind speed,${\;V}_{d}$ = 0.0009 m/s; R and $V_{d}$ refer to the value of [65]; $C_{h}$ is the hourly average PM_2.5_ concentration, μg/m^3^; $T_{c}$ is the area of forest, $T_{c}$ = 900 m^2^; and LAI is the leaf area index.

(2) Demand

This study expressed the demand for PR in terms of PM_2.5_ particles that exceed the WHO standard (affecting human health). The calculation formula is:

(15) $$D_{i}=\{\begin{array}{c} \left(C_{a}-{\mathrm{PM}}_{2.5\mathrm{permitted}}\right)\times H\times A\times 365\times 24,\;\;C_{a}>{\mathrm{PM}}_{2.5\mathrm{permitted}}\\ 0,\;\;C_{a}\leq {\mathrm{PM}}_{2.5\mathrm{permitted}} \end{array}$$

where $D_{i}$ is the demand for PR, μg; $C_{a}$ is the average annual PM_2.5_ concentration, μg/m^3^; ${\mathrm{PM}}_{2.5\mathrm{permitted}}$ is the standard threshold of average annual PM_2.5_ concentration stipulated by the World Health Organization [66], ${\mathrm{PM}}_{2.5\mathrm{permitted}}$ = 10 μg/m^3^; H is the height of the atmospheric boundary layer, H = 200 m [67]; and A is the pixel area, A = 900 m^2^.

#### 2.2.4. Flood Mitigation

(1) Supply

This study used the Urban Flood Risk Mitigation model of the InVEST model to calculate the supply of flood mitigation (FM). This module refers to the SCS-CN model and uses the curve number method to estimate $R_{\mathrm{in}}$. The calculation is as follows:

(16) $${\mathrm{FM}}_{i}=1-\frac{R_{\mathrm{pi}}}{P}$$

(17) $$R_{\mathrm{pi}}=\{\begin{array}{c} \frac{{\left(P-0.2I_{i}\right)}^{2}}{P+0.8I_{I}},\;\;\;P>0.2I_{i}\\ \;\;\;\;0,\;\;\;P\leq 0.2I_{i} \end{array}$$

(18) $$I_{i}=\frac{25400}{{\mathrm{CN}}_{i}}-254$$

where ${\mathrm{FM}}_{i}$ is the supply of FM on pixel i, which is dimensionless, with a value between 0 and 1; P is the depth of rainfall for the design storm, mm; $R_{\mathrm{pi}}$ is the runoff on pixel i, mm; $I_{i}$ is the potential retention on pixel i, mm; ${\mathrm{CN}}_{i}$ is the runoff curve value on pixel i, dimensionless; and ${\mathrm{CN}}_{i}$ refers to [68]; ${\mathrm{CN}}_{i}$ takes a value between 0 and 100. When ${\mathrm{CN}}_{i}$ is 100, it means that the rainfall is completely converted into runoff. The values of the coefficients for calculating FM are shown in Table 3.

(2) Demand

We refer to the research methods of [69,70] to quantify the demand of FM. The calculation is as follows:

(19) $${\mathrm{FRSD}}_{i}=x_{a}\times \left(a\times {\mathrm{PVI}}_{i}+b\times {\mathrm{EVI}}_{i}\right)$$

(20) $${\mathrm{PVI}}_{i}=x_{b}\times \left(\alpha \times {\mathrm{Pop}}_{i}+\beta \times {\mathrm{Old}}_{i}+\gamma \times {\mathrm{Child}}_{i}\right)$$

(21) $${\mathrm{EVI}}_{i}=x_{c}\times \left(\overset{n}{\underset{i=1}{\sum }}{\mathrm{EconomicScore}}_{i}\right)$$

where ${\mathrm{FRSD}}_{i}$ is the demand of FM on pixel i; ${\mathrm{PVI}}_{i}$ is the population vulnerability index on pixel i; ${\mathrm{EVI}}_{i}$ is the economic vulnerability index on pixel i, each LULC type has a corresponding score (Table 4); ${\mathrm{FRSD}}_{i}$, ${\mathrm{PVI}}_{i}$, anI ${\mathrm{EVI}}_{i}$ are dimensionless; a and b are the weights of the population vulnerability index and the economic vulnerability index, respectively, a = b = 1/2; $x_{a}$, $x_{b}{\;\mathrm{and}\;x}_{c}$ are Min-Max normalization coefficients representing the FR demand, PVI and EVI on pixel i, respectively. With this coefficient, FR demand, PVI and EVI can be normalized to a value between 0 and 1; ${\mathrm{Pop}}_{i}$ is the average population density on pixel i; ${\mathrm{Old}}_{i}$ is the population density of the elderly on pixel i; ${\mathrm{Child}}_{i}$ is the population density of children on pixel i; and α, β, and γ are the weights of the average population density, the population density of the elderly, and the population density of children, respectively, α = β = γ = 1/3.

#### 2.2.5. Heat Mitigation

(1) Supply

This study used the Urban Cooling model of the InVEST model to calculate the supply of heat mitigation (HM). The model utilizes shade, evapotranspiration, and albedo to calculate cooling capacity. The calculation is as follows:

(22) $${\mathrm{CC}}_{i}=0.6\times {\mathrm{shade}}_{i}+0.2\times {\mathrm{ETI}}_{i}+0.2\times {\mathrm{albedo}}_{i}$$

(23) $${\mathrm{ETI}}_{i}=\frac{K_{c}\times {\mathrm{ET}}_{0}}{{\mathrm{ET}}_{\max}}$$

(24) $${\mathrm{CC}}_{\mathrm{parki}}=\underset{j\in d\;\mathrm{radius}\;\mathrm{from}\;i}{\sum }g_{i}\times {\mathrm{CC}}_{j}\times e^{\left(\frac{-d\left(i,j\right)}{d_{\mathrm{cool}}}\right)}$$

(25) $${\mathrm{HM}}_{i}=\{\begin{array}{c} {\mathrm{CC}}_{i},\;\;\mathrm{if}\;i\;\mathrm{is}\;\mathrm{part}\;\mathrm{of}\;a\;\mathrm{large}\;\mathrm{green}\;\mathrm{area}\;\mathrm{or}\;{\mathrm{CC}}_{i}\geq {\mathrm{CC}}_{\mathrm{parki}}\\ {\mathrm{CC}}_{\mathrm{parki}},\;\;\mathrm{otherwise} \end{array}$$

where ${\mathrm{CC}}_{i}$ is the cooling capacity index on pixel i. Its value is comprised between 0 and 1, 0 means no cooling capacity, 1 means maximum cooling capacity; ${\mathrm{shade}}_{i}$ indicates the ability of trees to provide shade, set to 1 for trees taller than 2 m or 0 for trees below 2 m; ${\mathrm{ETI}}_{i}$ is the evapotranspiration index on pixel i; ${\mathrm{albedo}}_{i}$ represents the proportion of solar radiation reflected by the LULC type. Its value is between 0 and 1, where 0 represents the maximum absorption rate and 1 represents the maximum reflectivity. ${\mathrm{albedo}}_{i}$ refers to InVEST recommended data value [72]. ${\mathrm{ET}}_{0}$ is the potential evapotranspiration, mm; ${\mathrm{ET}}_{\max}$ is the maximum value of ${\mathrm{ET}}_{0}$ in the study area; $K_{c}$ is the evapotranspiration coefficient; $g_{i}$ indicates whether pixel i can be regarded as a green space. A value of 1 means LULC can be regarded as a green space, 0 means LULC cannot be regarded as a green space. ${\mathrm{shade}}_{i}$ and $g_{i}$ refer to the value of [73]; d(i,j) is the distance between pixel i and pixel j; $d_{\mathrm{cool}}$ is the distance for which a green space has a cooling effect; j∈d radius from i is the set of pixels whose distance to i is less than $d_{\mathrm{cool}}$; and ${\mathrm{HM}}_{i}$ is the demand for HM on pixel i. The specific coefficient values are shown in Table 5.

(2) Demand

We used the urban heat island effect intensity to express the demand for HM [74]. The calculation formula is:

(26) $$D_{i}=\{\begin{array}{cc} T_{i}-\frac{1}{n}\sum_{j=1}^{n}T_{\mathrm{cj}}, & T_{i}<\frac{1}{n}\sum_{j=1}^{n}T_{\mathrm{cj}}\\ \;\;\;\;\;\;0, & T_{i}>\frac{1}{n}\sum_{j=1}^{n}T_{\mathrm{cj}} \end{array}$$

(27) $${\mathrm{HMD}}_{i}=x_{d}\times D_{i}$$

where ${\mathrm{HMD}}_{i}$ is the demand of HM on pixel i, which is dimensionless; $x_{d}$ is the Min-Max normalization coefficient representing the HM demand on pixel i. With this coefficient, the HM demand can be normalized to a value between 0 and 1, removing the units of supply and demand, and then we can further calculate the ESDR to measure the relationship between supply and demand of HM; $T_{i}$ is the land surface temperature on pixel i, °C; n is the total number of pixels of cultivated land; $T_{\mathrm{cj}}$ is the land surface temperature of cultivated land on pixel j, °C;

#### 2.2.6. Landscape Recreation

(1) Supply

In this paper, the supply of landscape recreation (LR) was reflected by the green area, including forest and grassland [57]. The calculation formula is:

(28) $$S_{r}=A_{\mathrm{greenspace},\mathrm{subdistrict}}/A_{\mathrm{subdistrict}}$$

where $S_{r}$ is the supply of LR, m^2^/m^2^; $A_{\mathrm{greenspace},\mathrm{subdistrict}}$ is the green area of each jurisdiction, m^2^; and $A_{\mathrm{subdistrist}}$ is the total area of each jurisdiction, m^2^.

(2) Demand

In this paper, the demand for LR was reflected by the product of population density and the per capita green area planned by the government. The calculation formula is:

(29) $$D_{r}=P_{\mathrm{pop}}\times A_{\mathrm{guided}\;\mathrm{greenspace}}$$

where $D_{r}$ is the demand for LR, m^2^/m^2^; $P_{\mathrm{pop}}$ is the population density, person/m^2^. $A_{\mathrm{guided}\;\mathrm{greenspace}}$ is the per capita green area planned by the government, $A_{\mathrm{guided}\;\mathrm{greenspace}}$ = 13 m^2^/person.

### 2.3. Relationship between Supply and Demand of ESs

#### 2.3.1. Supply and Demand Relationship

This paper used the ecological supply–demand ratio (ESDR) to reflect the supply and demand relationship of ES, which may be surplus, balance, or deficit [75]. The calculation formula is:

(30) $$\mathrm{ESDR}=\frac{S-D}{\left(S_{\max}+D_{\max}\right)/2}$$

where S and D represent the supply and demand of ES, respectively; $S_{\max}$ and $D_{\max}$ represent the maximum supply and demand of ES, respectively. ESDR is expressed as a surplus (supply > demand) when it is completely greater than the range of 0. ESDR is expressed as a deficit(supply < demand) when it is completely less than the range of 0. ESDR is expressed as a balance (supply ≈ demand) when the range is close to 0. The higher the absolute value of ESDR, the greater the supply or demand for ESs.

#### 2.3.2. Identifying ES Budget Bundles

We clustered the ESDR using k-means cluster analysis in Geoda spatial analysis software. The principle of clustering is that the same ES budget bundle has the greatest similarity, and the difference between different clusters is the largest. According to the elbow method [40], the optimal number of clusters was determined to be 6, and ES budget bundles at the county, township, and village scales were obtained. Before clustering analysis, the Min–Max normalization method was used to unify the ESDR values into the range of [−1, 1] to reduce the influence of unit and size among ESs. The calculation formula is:

(31) $$x_{\mathrm{new}}=\frac{x-x_{\min}}{x_{\max}-x_{\min}}$$

where $x_{\mathrm{new}}$ is the result after normalization; $x_{\max}$ and $x_{\min}$ are the maximum and minimum values of sample x, respectively.

### 2.4. Driver Analysis of ES Budget Bundles

Referring to the research of [44,47,76,77,78] and combining it with the actual situation in the Su-Xi-Chang region, we select 14 driving factors that may affect the formation of ES budget bundles, including socioeconomic factors and natural environment factors. (1) Socioeconomic factors include GDP, population density (POP), percentage of impervious surface in 2020 (IS), and new impervious surfaces from 2015 to 2020 (NIS). (2) Natural environment factors: climatic factors include annual average precipitation (PRE), annual average temperature (TEM), annual average wind speed (WS), and annual average solar radiation (SR); soil factors include the percentage of sand in the soil (SAND), percentage of silt in the soil (SILT), and percentage of clay in the soil (CLAY); topographic factors include DEM, slope (SLOPE), and ground roughness (GR).

This paper employed the factor detector in Geodetector to identify the driving factors for the formation and spatial distribution of ES budget bundles [79]. The factor detector analyzes the degree of explanation of the independent variable X to the dependent variable Y. The calculation formula is:

(32) $$q=1-\frac{\sum_{h=1}^{L}N_{h}\sigma_{h}^{2}}{{N\sigma }^{2}}$$

where h=1,…,L is a layer of Y or X; $N_{h}$ and N are the number of units in the layer h and the whole area, respectively; $\sigma_{h}^{2}$ and $\sigma^{2}$ are the variance of the layer h and the whole area, respectively; q is the degree of explanation of the independent variable X to the dependent variable Y—that is, X explains q% of Y. The value of q is between 0 and 1; 0 means that there is no correlation, and 1 means that Y is completely determined by X.

## 3. Result

### 3.1. Multiscale Pattern Characteristics of ES Supply and Demand

#### 3.1.1. Crop Production

**Figure 2:**
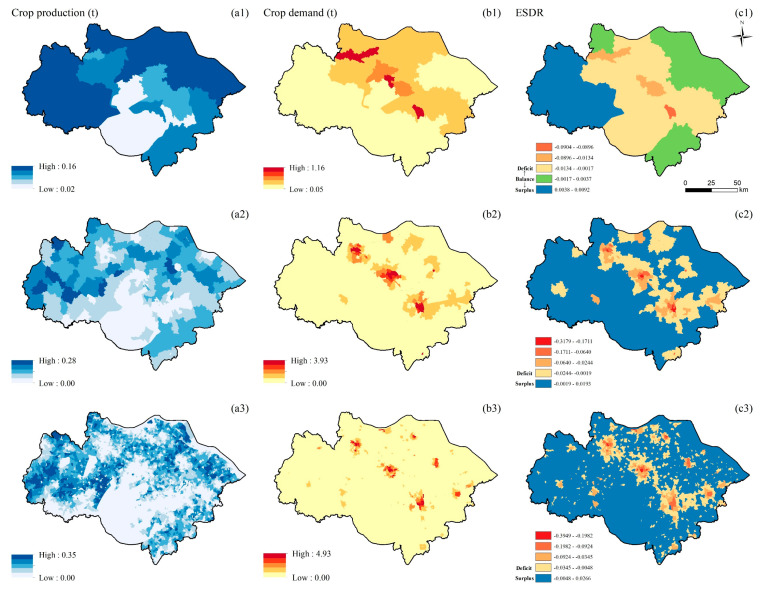
Spatial distribution of CP (unit: t/900 m^2^) at the county (**a1**,**b1**,**c1**), township (**a2**,**b2**,**c2**), and village (**a3**,**b3**,**c3**) scales; supply of CP (**a1**–**a3**); demand for CP (**b1**–**b3**); ESDR (**c1**–**c3**).

The supply of CP is spatially clustered on all three scales. The supply of CP at the township and village scales is similar to the distribution of cultivated land, and the high-value areas are concentrated in the west and northeast of the study area (Figure 2(a2,a3)). As the scale becomes larger, the high-value areas of supply increase (Figure 2(a1)). The distribution of CP demand at the township and village scales are similar, with a clustered distribution, and randomly distributed at the county scale. The high-value areas of CP demand are concentrated in the core areas of Suzhou, Wuxi, and Changzhou, and gradually decrease to the periphery (Figure 2(b1–b3)). The ESDR of CP is clustered at the township and village scales. At the township and village scales, deficit areas are concentrated in the urban core and surrounding areas, while other areas are in surplus (Figure 2(c2,c3)). The ESDR of CP is randomly distributed at the county scale. The deficit areas increased at the county scale, the high-value areas are concentrated in the urban core areas, and the surplus is located in the western part of the study area (Figure 2(c1)). As the scale grew, CP in the study area’s northeast, south, and northwest changed from surplus to balance.

#### 3.1.2. Water Retention

**Figure 3:**
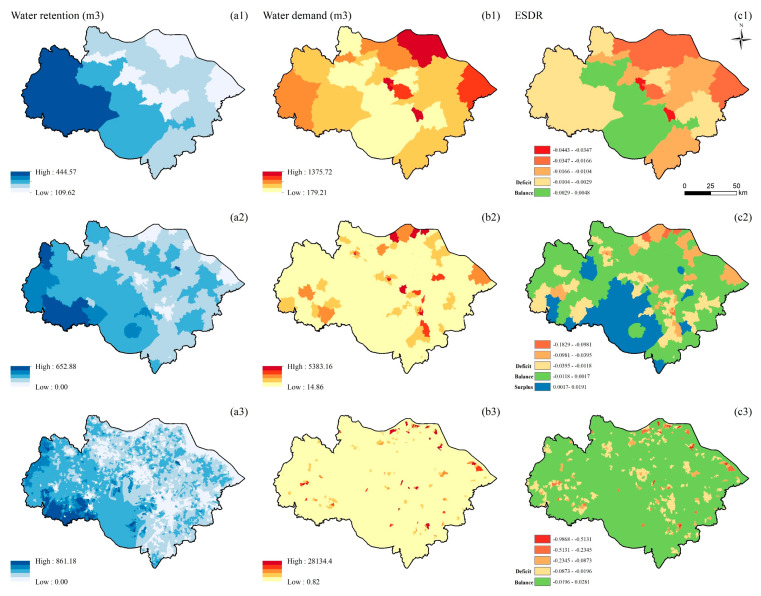
Spatial distribution of WR (unit: m^3^/900 m^2^) at the county (**a1**,**b1**,**c1**), township (**a2**,**b2**,**c2**), and village (**a3**,**b3**,**c3**) scales; supply of WR (**a1**–**a3**); demand for WR (**b1**–**b3**); ESDR (**c1**–**c3**).

The supply of WR has a similar spatial distribution pattern at the three scales. The high-value supply areas are distributed in the western part of the study area, followed by the southern part. The areas with the smallest supply are distributed in the northeastern and central parts of the study area (Figure 3(a1–a3)). The demand for WR is spatially clustered at the township and village scales. The high-value demand areas are distributed in the study area’s central, southwestern, and northeastern parts (Figure 3(b2,b3)). The demand for WR is randomly distributed at the county scale. As the scale becomes larger, the areas of higher demand gradually increase (Figure 3(b1)). The ESDR of WR is clustered at the township and village scales and randomly distributed at the county scale. At the village scale, the deficit and high-demand areas are distributed similarly, and supply and demand are balanced in the remaining areas (Figure 3(c3)). At the township scale, the southern part of the study area gradually turned from balance at the village scale to surplus. The high-value areas of the deficit are concentrated in the northeastern part of the study area (Figure 3(c2)), that is, along the Yangtze River, where there are many factories with high water demands. The deficit areas increase at the county scale and are distributed in the study area’s northeastern, western, and southern parts. High-value deficit areas are concentrated in urban core areas. The areas where supply and demand are balanced are distributed in the central and southern parts of the study area (Figure 3(c1)).

#### 3.1.3. PM_2.5_ Reduction

**Figure 4:**
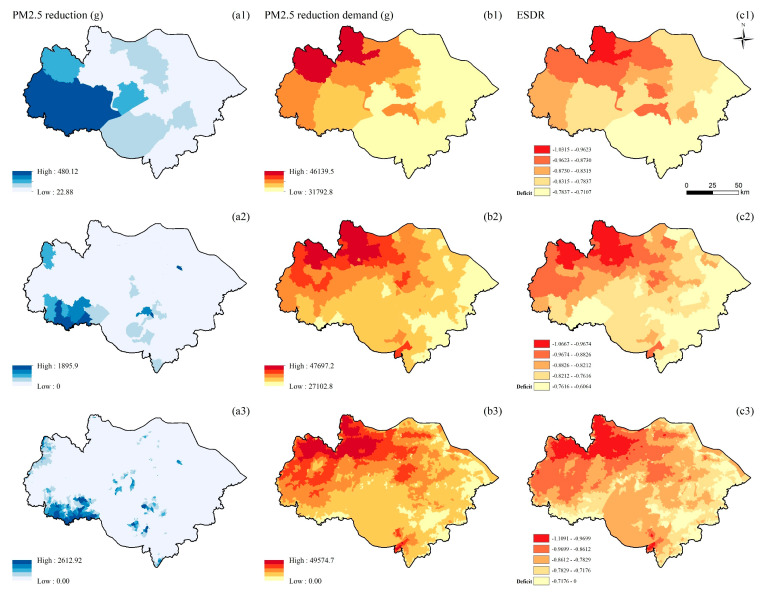
Spatial distribution of PR (unit: g/900 m^2^) at the county (**a1**,**b1**,**c1**), township (**a2**,**b2**,**c2**), and village (**a3**,**b3**,**c3**) scales; supply of PR (**a1**–**a3**); demand of PR (**b1**–**b3**); ESDR (**c1**–**c3**).

The supply and demand of PR have significant spatial agglomeration at the county, township, and village scales. The high-value supply areas on the three scales are concentrated in the west and south of the study area (Figure 4(a1–a3)). As the scale becomes larger, the high-value areas of supply increase gradually. The demand for PR gradually increases from the southeast to the northwest of the study area (Figure 4(b1–b3)). As the scale becomes larger, the high-value demand areas gradually decrease and are concentrated northwest of the study area. Although the southwest of the study area provides some supply of PR, the spatial pattern of ESDR and demand is similar because of the large demand. The PR of the entire study area is in deficit on all three scales. The high-value deficit areas are concentrated in the northwest of the study area, showing a downward trend from northwest to southeast (Figure 4(c1–c3)).

#### 3.1.4. Flood Mitigation

**Figure 5:**
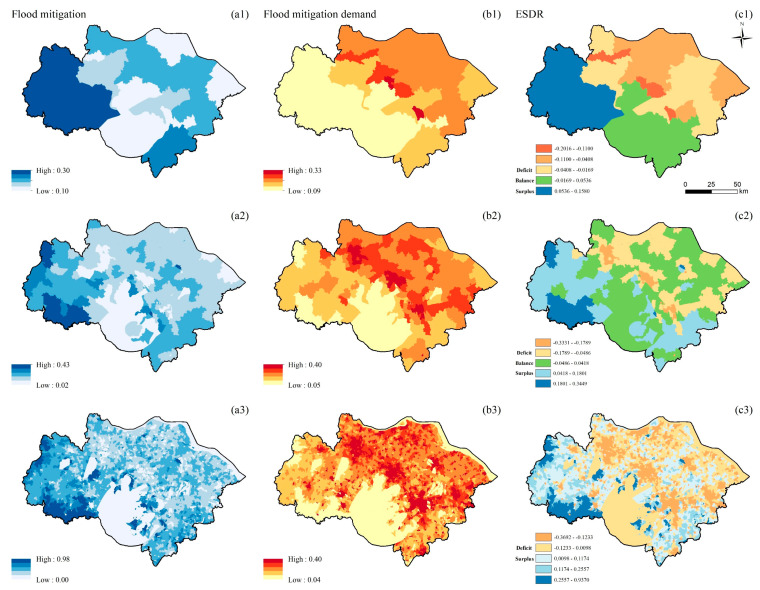
Spatial distribution of FM (unit: dimensionless, the value is between 0–1) at the county (**a1**,**b1**,**c1**), township (**a2**,**b2**,**c2**), and village (**a3**,**b3**,**c3**) scales; supply of FM (**a1**–**a3**); demand for FM (**b1**–**b3**); ESDR (**c1**–**c3**).

The high-value areas of FM supply at the three scales are distributed in the west, south, and north of the study area, with spatial aggregation. The distribution of FM demand at the township and village scales is similar, with a clustered distribution (Figure 5(b2,b3)). The demand for FM is randomly distributed at the county scale, with high-value areas distributed in the central and northeastern parts of the study area (Figure 5(b1)). The ESDR of FM is clustered at the three scales. The high-deficit areas of FM are concentrated in the urban core (Figure 5(c1–c3)). At the county scale, the western part of the study area is in surplus, the southern part is in balance, and the northeastern part is in deficit (Figure 5(c1)). There are many surplus and balance areas at the township scale (Figure 5(c2)). At the village scale, the western and southeastern parts of the study area are in surplus, and other areas are in deficit (Figure 5(c3)).

#### 3.1.5. Heat Mitigation

**Figure 6:**
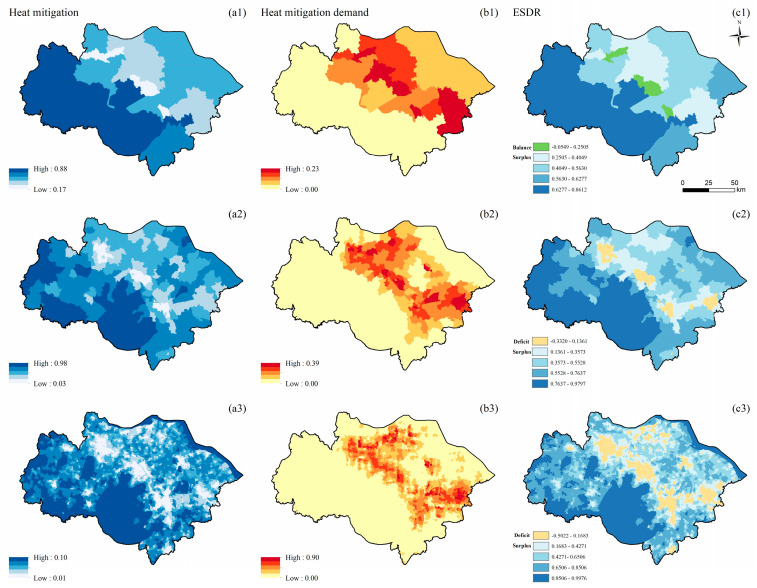
Spatial distribution of HM (unit: dimensionless, the value is between 0–1) at the county (**a1**,**b1**,**c1**), township (**a2**,**b2**,**c2**), and village (**a3**,**b3**,**c3**) scales; supply of HM (**a1**–**a3**); demand for HM (**b1**–**b3**); ESDR (**c1**–**c3**).

The supply of HM is clustered at the township and village scales and randomly distributed at the county scale. The high-value supply areas are mainly distributed in the west and south of the study area. The low-value areas are located in the core areas and the surrounding areas of Suzhou, Wuxi, and Changzhou (Figure 6(a1–a3)). The demand for HM is spatially clustered at three scales. Demand in the northeast of the study area is higher than in the southwest of the study area. The high-value demand areas are concentrated in the urban core areas (Figure 6(b1–b3)). The surplus level of HM in the west and southwest of the study area is optimal at the three scales. At the village scale, deficit areas are concentrated in the urban core and surrounding areas (Figure 6(c3)). The spatial distribution characteristics of ESDR also change as the scale becomes larger. For example, at the township scale, the HM deficit area decreases (Figure 6(c2)); at the county scale, the deficit area is transformed into balance (Figure 6(c1)).

#### 3.1.6. Landscape Recreation

**Figure 7:**
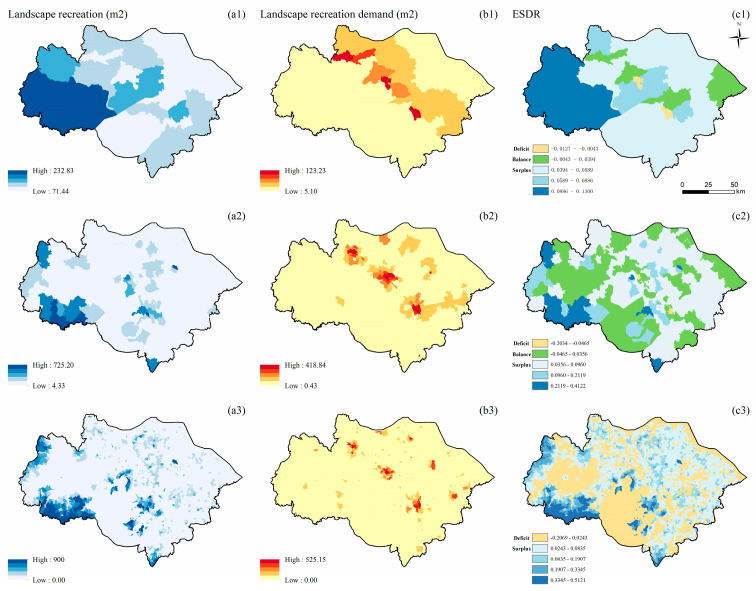
Spatial distribution of LR (unit: m^2^/900 m^2^) at the county (**a1**,**b1**,**c1**), township (**a2**,**b2**,**c2**), and village (**a3**,**b3**,**c3**) scales; supply of LR (**a1**–**a3**); demand for LR (**b1**–**b3**); ESDR (**c1**–**c3**).

The supply and demand of LR are randomly distributed at the county scale and clustered at the township and village scales. The supply of LR is mainly located in the west of the study area and along Taihu Lake. As the scale becomes larger, the high-value areas of supply increase gradually (Figure 7(a1–a3)). The high-value areas of demand for LR are concentrated in the urban core and surrounding areas (Figure 7(b1–b3)). At the county scale, the high-value areas of demand are concentrated in the core areas of Suzhou, Wuxi, and Changzhou (Figure 7(b1)). The ESDR of LR at the county scale is randomly distributed. Most areas at the county scale are in surplus and balance. The high-value areas of surplus are concentrated in Changzhou. The deficit areas are concentrated in the core areas of Suzhou and Wuxi (Figure 7(c1)). The value of ESDR also changes as the scale becomes smaller. For example, some areas at the township scale change from surplus to balance (Figure 7(c2)), and many areas at the village scale change from balance to deficit (Figure 7(c3)).

### 3.2. Multiscale Pattern Characteristics of ES Budget Bundles

**Figure 8:**
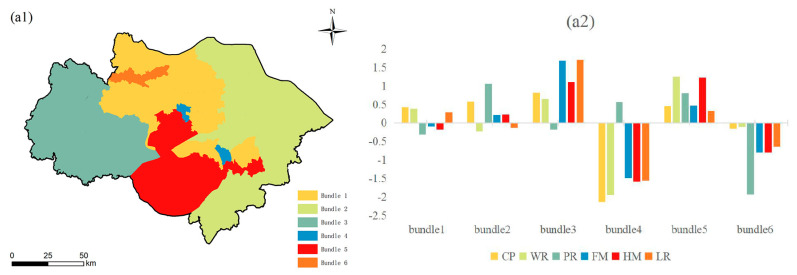
(**a1**) Spatial distribution of ES budget bundles at the county scale; (**a2**) ESDR of ES budget bundles at the county scale. Note: The histogram is obtained by the Z-score normalization method. The abscissa represents the six ES budget bundles on the county scale.; the vertical axis is the Z-score. When the Z-score = 0, it represents the average value of ESDR. A Z-score > 0 indicates above-average values, and a Z-score < 0 indicates below-average values.

**Figure 9:**
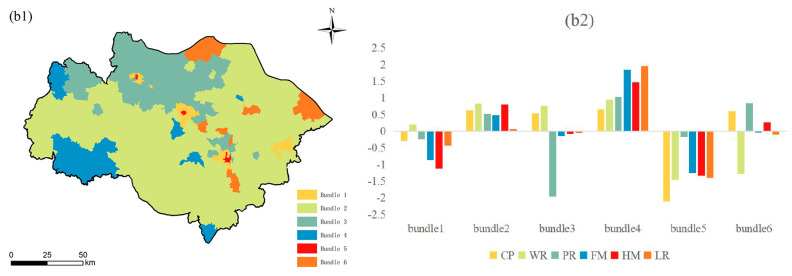
Spatial distribution of ES budget bundles at the township scale (**b1**); ESDR of ES budget bundles at the township scale (**b2**). Note: The histogram is obtained by the Z-score normalization method. The abscissa represents the six ES budget bundles on the township scale; the vertical axis is the Z-score. When Z-score = 0, it represents the average value of ESDR. Z-score > 0 indicates above-average values, and Z-score < 0 indicates below-average values.

**Figure 10:**
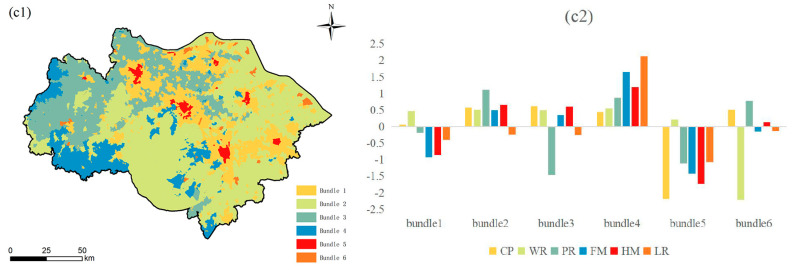
Spatial distribution of ES budget bundles at the village scale (**c1**); ESDR of ES budget bundles at the village scale (**c2**). Note: The histogram is obtained by the Z-score normalization method. The abscissa represents the six ES budget bundles at the village scale; the vertical axis is the Z-score. When Z-score = 0, it represents the average value of ESDR. Z-score > 0 indicates above-average values, and Z-score < 0 indicates below-average values.

Based on K-means clustering analysis, this study identified six ES budget bundles at the county (Figure 8), township (Figure 9), and village scales (Figure 10). Each bundle has similar ES characteristics [38]. This study found that ES budget bundles at the township and village scales have similar spatial patterns. However, compared with the township scale, the village scale is finer and more similar to the spatial distribution of LULC. However, the distribution of ES budget bundles at the township/village scale and the county scale are significantly different. The spatial distribution of ES budget bundles at the county scale is similar to that of municipal administrative districts, forming the spatial pattern of Suzhou, Wuxi, Changzhou, Taihu Lake and its surrounding areas, and urban core areas.

#### 3.2.1. County Scale

Bundle 1 is mainly distributed in the north of Wuxi and Changzhou, accounting for 23.3% of the total study area. CP, WR, and LR are slightly higher than the mean value of the study area; PR, HM, and FM are slightly lower than the mean value of the study area. Bundle 2 is distributed in the relatively flat eastern region, concentrated in Suzhou, accounting for 32.44% of the entire study area. Bundle 2 has the highest level of PR and relatively high CP. FM and HM are slightly above the mean values. WR and LR are below the mean. Bundle 3 is distributed in the slightly higher western region, concentrated in the south of Wuxi and Changzhou, accounting for 25.55% of the total study area. All ESs are above the mean value of the study area, except for PR, which is slightly below average. FM and LR are at the highest level in the study area. Bundle 4 is distributed in the middle of the study area, accounting for 0.88% of the total study area. Bundle 4 is concentrated in the urban core areas of Wuxi and Suzhou. Due to the high demand, CP, WR, HM, LR, and FM are all lower than the average value of the study area, except for the PR, which is slightly higher than the average value. Bundle 5 is distributed in the Taihu Lake Scenic Area in the south of the study area, accounting for 16.2% of the total study area. The six ESs are all higher than the mean value, especially WR and HM, which are at the highest level in the study area. Bundle 6 is distributed in the northwest of the study area, located in the core area of Changzhou, accounting for 1.63% of the total study area. The six ESs in bundle 6 are all below the mean value, especially PR, which is at the lowest level in the study area.

#### 3.2.2. Township Scale

Bundle 1 is distributed in the surrounding areas of the urban core areas of Suzhou, Wuxi, and Changzhou, accounting for 2.21% of the total study area. All ESs in bundle 1 have below-average values, except for WR, which is slightly above the mean value of the study area. Bundle 2 has the largest area at the three scales, accounting for 65.77% of the total study area. LR is at the average level in the study area, and the remaining five ESs are above the mean value. Bundle 3 is distributed in the relatively flat northern area, accounting for 19.07% of the total study area. Although WR and CP are above average, PR is at the lowest level in the study area. In addition, FM, HM, and LR are the average levels of the study area. Bundle 4 is mainly distributed in the southwest of the study area, accounting for 8.24% of the total study area. The high supply and low demand in the area where bundle 4 is located make all of the six ESs in bundle 4 at the highest level in the study area, especially LR, FM, and HM. Bundle 5 is concentrated in the urban core areas of Suzhou, Wuxi, and Changzhou, accounting for 0.19% of the total study area. The six ESs in this region are all below the average, and CP is at the lowest level in the study area. Bundle 6 is distributed in the study area’s northern, northeastern, and central parts, accounting for 4.52% of the total study area. WR in bundle 6 is at a lower level; PR, CP, and HM are higher than the average of the study area; and LR and FM are at the average level of the study area.

#### 3.2.3. Village Scale

The LULC types of bundle 1 are mainly impervious surface (57.7%) and cultivated land (17.6%) (the area proportion of LULC types in ES budget bundles at the village scale was obtained through the zoning statistical tool of ArcGIS 10.8), accounting for 19.93% of the total study area. The HM, FM, LR, and PR of bundle 1 are lower than the average value of the study area; WR is higher than the average value of the study area; CP is the average level of the study area. Bundle 2 is distributed in the central and eastern parts of the study area, accounting for 45.91% of the total study area. The LULC types of bundle 2 are mainly water (44.76%) and cultivated land (30.21%). All ESs are above-average values except for LR, which is slightly below the mean value of the study area. In particular, PR is at the optimal level in the study area. Bundle 3 is located in the west and northwest of the study area, accounting for 21.4% of the total study area. These areas are mainly covered by cultivated land (54.94%) and impervious surfaces (18.21%). The PR of bundle 3 is much lower than the average of the study area; the LR is slightly lower than the average of the study area; and the CP, HM, WR, and FM are slightly higher than the average. Bundle 4 is distributed in the forest (64.5%) in the western and central parts of the study area. The areas of cultivated land (17.22%) and impervious surface (2.28%) in bundle 4 were smaller and less disturbed by human activities. Thus, bundle 4 provides the highest LR, FM, HM, and more PR levels in the study area. However, its area only accounts for 10.19% of the study area. Bundle 5 is mainly concentrated in the urban core areas of Suzhou, Wuxi, and Changzhou, accounting for 1.61% of the study area. CP, HM, FM, and LR are all at the lowest levels in the study area, except for WR, which is slightly above average. Bundle 6 is distributed in the northeastern part of the study area, accounting for 0.96% of the total study area. LULC types of bundle 6 are mainly cultivated land (33.55%) and industrial land (26.85%). WR in bundle 6 has the lowest level in the study area; PR and CP have above-average values; and FM, HM, and LR have the average level of the study area.

### 3.3. Driver Analysis of ES Budget Bundles

This study analyzed the extent to which different drivers explain the formation of ES budget bundles using the factor detector in Geodetector. Table 6 shows the results of the factor detection at the county, township, and village scales. (1) County scale: POP (0.729531) > IS (0.630183). (2) Township scale: POP (0.286231) > IS (0.220963) > GDP (0.158807) > NIS (0.153661) > SR (0.144641) > DEM (0.139593) > TEM (0.114315) > CLAY (0.085909) > SAND (0.083865) > SILT (0.073237). (3) Village scale: POP (0.331799) > IS (0.186227) > DEM (0.105744) > SLOPE (0.097106) > SR (0.087445) > GR (0.077787) > GDP (0.060104) > PRE (0.054848) > TEM (0.026132) > NIS (0.024559) > SILT (0.018906) > WS (0.012526) > CLAY (0.007144) > SAND (0.005382).

## 4. Discussion

This paper analyzed the supply, demand, and budgets of ESs and the drivers of ES budget bundles at the county, township, and village scales in the Su-Xi-Chang region. Unlike previous studies, this study focused on multiscale analysis and analyzed the impact of multiple drivers on ES budget bundles in rapidly urbanizing regions. The issues we attempted to address were threefold. (1) Theoretical implications: to determine the characteristics of the multiscale pattern of ES supply and demand in the Su-Xi-Chang region and to analyze the impact of the natural environment and socioeconomic factors on ES budget bundles. (2) Practical implications: to propose a multiscale decision-making process. (3) To discuss the limitations and future perspectives of this study.

### 4.1. Multiscale Pattern of Supply and Demand of ESs

This paper evaluated the supply and demand patterns of six typical ESs at the county, township, and village scales in the Su-Xi-Chang region and identifies six ES budget bundles. The results showed that the supply, demand, and budgets of ES are scale-dependent, which is consistent with the findings of [25]. On the one hand, the large scale masks the spatial heterogeneity and clustering characteristics of the supply and demand distribution of ESs at the small scale, similar to the findings of [58]. In this study, the distribution of supply and demand of ESs was more refined at the township and village scales than at the county scale. In addition, the demand for CP, WR, FM, and LR, as well as the supply of HM and LR, were randomly distributed at the county scale but clustered at the township and village scales. Therefore, it is unscientific to simply add, generalize, or extrapolate the information represented by a certain scale. On the other hand, the large scale highlights high-value areas of supply and demand. In this study, the high-value areas of CP supply were concentrated in cultivated land. The high-value areas of supply of regulating services (WR, PR, HM, and FM) and cultural service (LR) were concentrated in the forest and water areas. The high-value areas of demand were mainly concentrated in the urban core areas.

Due to the inconsistency in the quantity and location of the supply and demand of various ESs, all ESs at the three scales showed a mismatch between supply and demand. The position and area of surplus, balance, and deficit vary with scale. For example, the supply of HM at the county scale can meet the demand, but the township and village scales cannot (Figure 5(c1–c3)). On the three scales, CP, PR, and FM all showed a deficit in the urban core areas, unable to meet their own needs through local supply. ES budget bundles also exhibited scale dependence. The spatial patterns and inter-service composition of ES budget bundles at the township and village scales were similar (Figure 7 and Figure 8), while there were significant differences between the township/village scale and the county scale (Figure 6). This is similar to the findings of a study in Quebec, Canada [25], which demonstrated that the spatial pattern and inter-service composition of ES supply bundles were similar at the two smaller scales (1 km and 3 km) but very different at a larger scale (9 km). This suggests that the formation of ESB at similar scales is similar and stable. When the scale becomes larger or smaller, the spatial pattern and the inter-service composition of ESB will be reconfigured. In summary, we believe that the county scale in the Su-Xi-Chang region is suitable for analyzing the overall situation of ESs, while the township and village scales include detailed information and are suitable for implementing precise management.

### 4.2. The Impact of the Natural Environment and Socioeconomics on ES Budget Bundles

Clarifying the reasons for the formation of ESB will help to deepen the understanding of the coupling between the natural environment and the social economy and inform managers of the underlying mechanisms that affect decision-making [41]. In this study, social factors (POP and IS) determined the formation of ES budget bundles at three scales (Table 6), which is similar to the findings of [42,76]. Economic factors (GDP) only played a significant role at the township scale, which is consistent with the findings of [3]. Natural environment factors (such as DEM, SR, and PRE) played a role at the township and village scales. Our results show that, compared with natural environmental factors, socioeconomic factors play a dominant role in forming ES budget bundles in the Su-Xi-Chang region.

We found that the drivers of ES budget bundles were also scale-dependent (Table 6). The larger the scale, the stronger the explanation of the driving factors, similar to the results of a study in the Yangtze River Delta region [56]. The larger the scale, the more single the driving factor, and the smaller the scale, the more diverse the driving factor, which is consistent with the research results of [3]. While small scales are better for identifying drivers [80], multiscale analysis of drivers of ESBs in decision-making helps to capture the key at different scales [3].

Therefore, landscape planners and managers must comprehensively consider local natural conditions, typical ES, and scales when making decisions [41]. On the other hand, it is also necessary to focus on regulating human activities [45]. Increasing supply will undoubtedly improve the mismatch between supply and demand of ESs, but it is unrealistic to only increase supply without reducing demand to achieve the sustainable use of ESs [18]. It may be difficult to solve the mismatch between the supply and demand of various ESs at different scales [57], but it will bring about a mismatch or trade-off between the supply and demand of other services [56].

### 4.3. Multiscale Decision-Making Process

**Figure 11:**
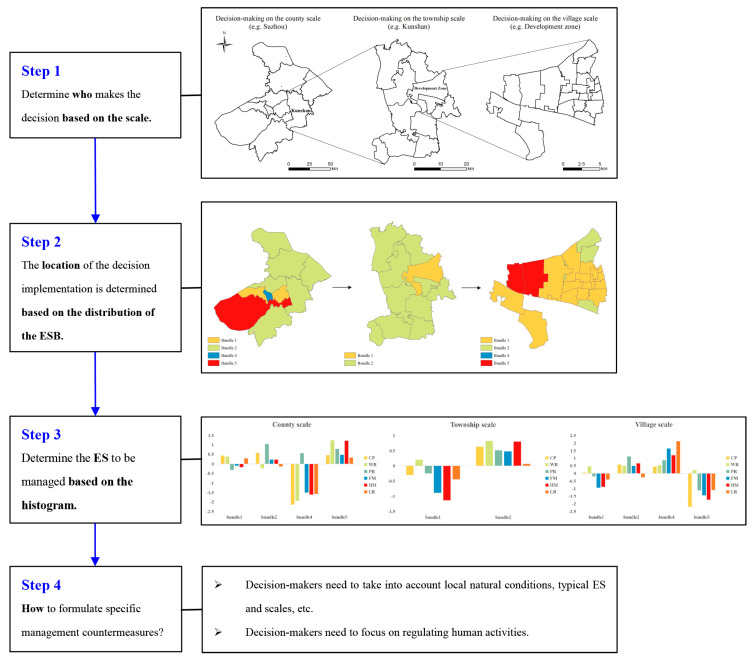
Four steps of the multiscale decision-making process (taking the Su-Xi-Chang region as an example).

We combined multiscale ES budget bundles and drivers to inform decision-making processes toward the sustainable use of ESs. The multiscale decision-making process consists of four steps. The first step is to determine who will govern based on the scale. The regions of Suzhou, Kunshan (the county-level administrative unit under Suzhou), and Kunshan development zone correspond to the management scope of managers at the county scale, the township scale, and the village scale, respectively (Figure 11, step 1). The second step is to determine where to implement the decision based on the distribution of ES budget bundles (Figure 11, step 2). The county and village scales include bundle 1, bundle 2, bundle 4, and bundle 5, and the township scale includes bundle 1 and bundle 2. The third step is to determine the ESs that need to be managed based on the histogram (Figure 11, step 3). The fourth step is to formulate specific management countermeasures based on drivers, local natural conditions, typical ESs, scale, and stakeholder preferences (Figure 11, step 4) [81].

In the example shown in Figure 11, the area of bundle 2 is the largest at the county scale, so the situation in the area where bundle 2 is located can best represent the overall situation of Suzhou. The PR and CP in bundle 2 are better, but the WR and LR are slightly lower than the mean value of the study area. Therefore, decision-makers at the county scale need to improve the WR and LR of the area where bundle 2 is located. When refining from the county scale to the township scale, Kunshan includes bundle 2, where all ESs are higher than the average level of the study area, and bundle 1, where all ESs except WR are lower than the mean value. Then, the decision-makers at the township scale need to include the region where bundle 1 is located in the decision-making scope and take the improvement of HM and FM as the main task. From the township scale to the village scale, bundle 1 and bundle 5 are the main ES budget bundles of the Kunshan Development Zone. The ESs in bundle 5 have more below-average values than the ESs in bundle 1, especially CP, HM, and FM. Therefore, when the workload is heavy, decision-makers can prioritize bundle 5 at the village scale as the priority area of management. Combined with the driving factors, policy-makers need to focus on regulating human activities and comprehensively consider local natural conditions, typical ES, and scale [45]. At the same time, cultivated land should be used intensively, and the conversion of other lands to urban land should be controlled [3]. Strengthening the cross-regional flow of ES, giving play to the leading role of the advantageous bundle, and complementing the disadvantaged bundle can help achieve a win–win situation [46].

In conclusion, decision-makers at the county scale should pay attention to the overall situation of ES and find the prominent locations of the imbalance between supply and demand. In this way, the decision-making time is shortened, decision-making efficiency is improved, and overall decisions that meet the interests of most people are made. Decision-makers at the township scale have clearer images and can make decisions that are more in line with local conditions, with decisions that are consistent with the higher-level scale. Managers at the village scale should incorporate the opinions of stakeholders and help decision-makers at the larger scale fill in the small but important issues that are easily overlooked. The large scale highlights the focus of decision-making, the small scale makes up for the lack of precision in the large scale, and the combination of multiple scales can make ES management more effective [24,37]. The multiscale decision-making process we propose can be broadly applied to making management decisions in other rapidly urbanizing regions of the world.

### 4.4. Limitation

(1) In terms of supply and demand assessment, from the perspective of demand, for example, the demand for WR is the actual demand calculated according to the statistical data. At the same time, the demand for LR was calculated based on the minimum threshold. Therefore, the actual demand for LR may be greater. From the perspective of supply, there is no distinction between actual supply and potential supply. The supply of ES evaluated by the InVEST model is the potential supply, such as FM, which belongs to the potential supply (maximum threshold). (2) It is necessary to map and analyze the supply and demand of ESs in the three administrative spatial units of county, township, and village. This helps to link production to consumption and also meets the decision-making needs of managers [76]. However, the range of influence of ESs is of many shapes and may be larger or smaller than the range of administrative boundaries [36]. In this study, most ESs were generated locally, but WR, for example, was delineated by watershed, and administrative boundaries may have split some ecological boundaries. Therefore, decision-makers need to work with surrounding areas to incorporate the actual management scope of ES into the decision-making process [82]. (3) We did not consider the ES flow, which may alleviate the supply–demand imbalance to a certain extent, which will be the direction of further research in the future.

## 5. Conclusions

This paper analyzed the supply, demand, and budgets of ESs and the drivers of ES budget bundles at the county, township, and village scales in the Su-Xi-Chang region. The results showed that the high-value areas of ES supply were mainly distributed in the western part of the study area. The high-value areas of ES demand were mainly concentrated in urban core areas. Due to the inconsistency in the quantity and location of the supply and demand of various ESs, ESs on the three scales all showed a mismatch between supply and demand. Among them, CP, PR, and FM showed deficits in the urban core area and could not meet their own needs through local supply. The location and area of ES supply and demand surpluses, balances, and deficits varied with scale. The spatial patterns of ES supply, ES demand, and ES budget bundles at the township and village scales were similar, while the township/village and county scales were different. We found that socioeconomic factors significantly impact ES budget bundles in the Su-Xi-Chang area more than natural environment factors. The top two drivers on the three scales were population density and the percentage of impervious surfaces. The diversity and degree of interpretation of influencing factors varied with scale. We believe that focusing on the big picture on a large scale, implementing precise management on a small scale, and focusing on regulating human activities can make management decisions more effective. This study can provide a scientific basis for the sustainable utilization of ecosystem services in the Su-Xi-Chang region, and the research results and methods can provide a reference for similar studies in other rapidly urbanizing areas in the world.

## Figures and Tables

**Table 1:** Data used in this study.

Data Name	Data Type	Year	Source
Chinese administrative boundaries	Vector data	2015	Resource and Environment Science and Data Center (https://www.resdc.cn/DataList.aspx accessed on 5 August 2022)
Administrative boundaries in the Su-Xi-Chang region	County, township, village. Vector data	2009	The Second National Land Survey
LULC	10 m × 10 m Raster data	2020	Earth Online (https://earth.esa.int/web/guest/home accessed on 18 May 2022)
Total population	County Statistical data	2020	Statistical yearbook of CNKI (https://data.cnki.net/Yearbook accessed on 7 May 2022)
Population density	100 m × 100 m Raster data	2020	WorldPop (https://www.worldpop.org accessed on 23 May 2022)
Normalized difference vegetation index	30 m × 30 m Raster data	2020	Resource and Environment Science and Data Center (https://www.resdc.cn/DataList.aspx accessed on 20 May 2022)
Soil depth, sand, silt, clay, soil organic matter content	1 km × 1 km Raster data	1995	Agriculture Organization of United Nations (https://www.fao.org/soils-portal/data-hub/soil-maps-and-databases/harmonized-world-soil-database-v12 accessed on 8 February 2022)
Average precipitation	1 km × 1 km Raster data	2020	National Earth System Science Data Center (http://www.geodata.cn accessed on 5 February 2022)
Potential evapotranspiration	1 km × 1 km Raster data	2020	National Earth System Science Data Center (http://www.geodata.cn accessed on 5 February 2022)
Water consumption	County Statistical data	2020	Water Resources Bulletin of Water Resources Bureau
Hydrologic soil groups	250 m × 250 m Raster data	2020	Global Hydrologic Soil Groups for Curve Number-Based Runoff Modeling (https://daac.ornl.gov/accessed on 6 May 2022)
PM_2.5_ concentration	1 km × 1 km Raster data	2020	National Earth System Science Data Center (http://www.geodata.cn accessed on 5 July 2022)
Leaf area index	500 m × 500 m Raster data	2019	National Earth System Science Data Center (http://www.geodata.cn accessed on 18 May 2022)
Land surface temperature	1 km × 1 km Raster data	2020	Institute of Tibetan Plateau Research, Chinese Academy of Sciences (http://data.tpdc.ac.cn accessed on 8 June 2022)
GDP	1 km × 1 km Raster data	2019	Resource and Environment Science and Data Center (https://www.resdc.cn/DataList.aspx accessed on 4 July 2022)
New impervious surfaces	30 m × 30 m Raster data	2015–2020	https://doi.org/10.5281/zenodo.5220816 [59]
DEM	30 m × 30 m Raster data	2020	Geospatial Data Cloud (http://www.gscloud.cn/search accessed on 7 July 2022)
Slope, roughness	30 m × 30 m Raster data	2020	Obtained by DEM processing in ArcMap10.8 software.
Average temperature	1 km × 1 km Raster data	2020	National Earth System Science Data Center (http://www.geodata.cn accessed on 5 July 2022)
Average wind speed	1 km × 1 km Raster data	2020	National Earth System Science Data Center (http://www.geodata.cn accessed on 7 July 2022)
Solar radiation	300 m × 300 m Raster data	2007–2021	Global Solar Atlas (https://globalsolaratlas.info accessed on 8 July 2022)

**Table 2:** WR coefficient table.

LULC_Desc	Lucode	Kc	Root_Depth	LULC_Veg	C_j_
Cultivated land	1	0.6	1000	1	0.347
Forest	2	1	7000	1	0.0267
Grassland	3	0.65	1500	1	0.0937
Water area	4	1	1000	0	0
Built-up area	5	0.3	500	0	1
Unused land	6	0.2	10	0	1

Note: Kc is the evapotranspiration coefficient, root_depth is the maximum root depth for plants, and LULC_veg refers to whether there is vegetation on the LULC. A value of 1 means vegetation is present; 0 means no vegetation.

**Table 3:** FM coefficient table.

Lucode	LULC_Desc	CN_A	CN_B	CN_C	CN_D
1	Cultivated land	54	70	80	84
2	Forest	36	60	73	79
3	Grassland	49	69	79	84
4	Water area	0	0	0	0
5	Built-up area	85	90	92	94
6	Unused land	77	86	91	94

Note: CN_(A–D) is the curve number value of the LULC type in the hydrological soil group.

**Table 4:** Economic Vulnerability Score for each LULC type.

LULC_Desc	Economic Score
Developed, high density (a_ISA_ > 80%)	8
Developed, moderate density (50% < a_ISA_ ≤ 80%)	7
Developed, low density (20% < a_ISA_ ≤ 50%)	6
Developed, open land (a_ISA_ ≤ 20%)	5
Unused land	4
Cultivated land	3
Grassland	2
Forest/water area	1

Note: ${\mathrm{Economic}\;\mathrm{Score}}_{i}$ refers to [69,70,71]; a_ISA_ is the ratio of the impervious surface area to the pixel area (30 m × 30 m). The larger the value of a_ISA_, the denser the impervious surface on pixel i, and the higher the economic vulnerability.

**Table 5:** HM coefficient table.

Lucode	Shade	Kc	Albedo	g_i_
1	0	0.6	0.2	1
2	1	1	0.2	1
3	0	0.65	0.2	1
4	0	1	0.05	0
5	0	0.3	0.15	0
6	0	0.2	0.25	0

**Table 6:** Results of factor detection.

	County Scale		Township Scale		Village Scale	
Variable	q-Statistic	*p*-Value	q-Statistic	*p*-Value	q-Statistic	*p*-Value
GDP	0.352705		0.158807	**	0.060104	**
POP	0.729531	**	0.286231	**	0.331799	**
IS	0.630183	**	0.220963	**	0.186227	**
NIS	0.509151		0.153661	**	0.024559	**
PRE	0.381515		0.024387		0.054848	**
TEM	0.285643		0.114315	**	0.026132	**
WS	0.509074		0.017601		0.012526	**
SR	0.329032		0.144641	**	0.087445	**
SAND	0.308795		0.083865	**	0.005382	**
SILT	0.201164		0.073237	**	0.018906	**
CLAY	0.140203		0.085909	**	0.007144	**
DEM	0.150341		0.139593	**	0.105744	**
SLOPE	0.275312		0.044057		0.097106	**
GR	0.374718		0.050881		0.077787	**

Note: The larger the q-value, the stronger the interpretation of X by Y. The q-value means that X explains 100 × q% of Y; the *p*-value indicates the degree of significance. *p* < 0.1 indicates significance, and it is indicated by **.

## Data Availability

All data generated or analyzed during this study are included in the published article.
